# Testing the generalized validity of the Emotion Knowledge test scores

**DOI:** 10.1371/journal.pone.0207335

**Published:** 2018-11-14

**Authors:** Ana R. Delgado, Debora I. Burin, Gerardo Prieto

**Affiliations:** 1 Facultad de Psicología, Universidad de Salamanca, Salamanca, Spain; 2 Facultad de Psicología, Universidad de Buenos Aires-CONICET, Buenos Aires, Argentina; University of Copenhagen, DENMARK

## Abstract

Differential item functioning **(**DIF) is of the utmost importance in order to corroborate the generalized validity of test scores in different groups. DIF indicates that an item does not function equally in different groups such as age, gender or cultural ones. Our objective was to contrast the generalized validity of the Emotion Knowledge (EK) test scores in a heterogeneous Argentinian sample composed of 100 females and 100 males (age range: 18–65). Data from the original validation sample (200 Spanish participants, half of them males) were conjointly analyzed (total n = 400). Results of the Rasch Model (RM) analysis indicated that both fit to the RM and reliability (*ISR* = .97, *PSR* = .80) were adequate. Item *logit* measures ranged from -3.89 to 3.68, and person *logit* measures ranged from -1.12 to 5.09, with a mean value of 2.36. DIF was tested for gender, age, educational level and country, with a few item contrasts found to be statistically significant. Even though small significant differences in EK scores were associated with educational level (*d* = .25) and country (*d* = -.25), they became non-significant after removing the seven country-related DIF affected items. We can conclude that there is enough evidence for the generalized validity of EK test scores in Argentina. Given that recent theories of human emotion consider conceptual knowledge supported by language as *constitutive* of emotions, the EK test can be used in academic or applied settings where individual differences in emotional competence might be relevant.

## Introduction

Recent theories of human emotion consider conceptual knowledge supported by language as *constitutive* of emotions [1–5]. In this view, emotions are not modules in the brain that trigger fixed expressive responses [6], but constructed affective states, guided by categories and language. Previous constructionist approaches conceived of emotions as semantic scripts of prototypical behaviors, expressions, labels and words [7]. Developmentally, children would go from a broad, valence-based system to knowing full scripts for specific discrete categories of emotion [8]. Furthermore, for the conceptual act theory [4] emotional categories are not fixed scripts, but constructed mental phenomena anchored in concepts and language. Emotions, like the rest of mental life, emerge as a consequence of the human brain’s tendency to categorize, to make the contingencies meaningful. Different instances of sensory inputs, core affective states (valence, arousal), interactions, and behavior could be grouped together into the same category and given the same name. Some of these categories might be cross-culturally stable, whereas other categories are culture specific. Language plays a central role in this view: words are the “glue” that brings together different instances into a coherent category [2, 4]. Therefore, the conceptual act theory predicts general agreement within broad emotional categories for people using the same language, even though certain sub-cultural differences in Emotion Knowledge (EK) could be found. In the context of discrete emotion theories, EK has been defined as related to the *understanding* of discrete emotions and differentiated from semantically close concepts such as emotion utilization (the adaptive use of emotion arousal) and emotion regulation [9].

The construction and validation of EK tests is of interest both from the theoretical and the applied points of view. The most-used emotional intelligence test is the Mayer Salovey Caruso Emotional Intelligence Test (MSCEIT), although only one of its facets, that of *understanding*, has received enough empirical support as a measure of aptitude [10]. Mayer, Salovey and Caruso [11] have clarified their original description of the understanding area of the MSCEIT: " […] we meant that a person who possessed emotional knowledge could understand emotional word meanings and concepts, understand the situations […]" (p. 404). They have recently described Emotional Intelligence as one of the broad intelligences in the context of a hierarchical model that empirically categorizes human abilities into areas such as fluid reasoning, visual spatial processing or *comprehension-knowledge*, considering that if emotional intelligence is really a discrete intelligence, it would be needed to make the case that there has evolved a separate reasoning capacity to *understand* emotions [12]. In addition to the relevance that Mayer, Salovey and Caruso attribute to EK [11, 12], emotional competence test scores predict various socially relevant outcomes [13–15].

The reasons summarized above led to the construction of language-based EK tests [16] by means of the Rasch Model (RM), an implementation of the invariant measurement approach [17–22]. The RM indicates that the probability that person *n* passes item *i* is Pni = exp(Bn-Di)/(1+exp[Bn-Di]), Bn: person level, Di: item location. If the empirical data fit the model adequately, then person measures and item locations can be jointly measured on an *interval* scale in *logit* units. Evidence of unidimensionality was found when scaling the scores from the three EK tests conjointly [16] and so, for the purposes of this paper, we will refer to the EK *test*. In the invariant measurement realm, an important empirical testing of generalized validity can be carried out by testing the lack of Differential Item Functioning **(**DIF).

DIF indicates that an item measures differently in different contexts: item locations are not invariant across various groups, breaking the model requirement of person invariant calibration of test items [22]. It is unlikely to be detected at an individual level, and so it is usually checked for groups based on gender or culture to ensure test fairness [23]. DIF analysis tests the generalized validity of the measures for different groups. The usual procedure in the RM context is to test the standardized difference between item calibrations in two groups (i.e., Argentina and Spain, male and female, etc.) with Bonferroni-corrected *alpha* levels; the Rasch-modeled scores from the analysis of all the participants are held constant, providing the conjoint measurement scale in *logit* units [24, 25].

Thus, the objective of this study was to test the generalized validity of the Emotion Knowledge (EK) test scores, originally validated in Spain, with new data from an Argentinian sample. Our aim was achieved.

## Materials and methods

### Participants

The sample was composed of 100 females and 100 males, with ages ranging from 18 to 65 years old, Spanish as first language, and Argentinian nationality. Participants were recruited in public places (e.g., a coffee shop, a bus station, a gym) and psychology students were excluded from the sample. Inclusion criteria were similar to the original Spanish sample [16]. Roughly half of the participants (n = 93) were young adults (18–30). As to educational level, 101 participants were or had been to college or further. The Spanish data came from a sample that was demographically similar, except for the fact that the educational level was higher (155 subjects were or had been to college or further).

### Instruments

Evidence of unidimensionality for the total score was found in the process of constructing and validating the EK scores [16]. This is why the EK test can be described as composed of three subtests (the original tests: Emotion Vocabulary, Close Emotional Situations, Far Emotional Situations).

**Fig 1:**
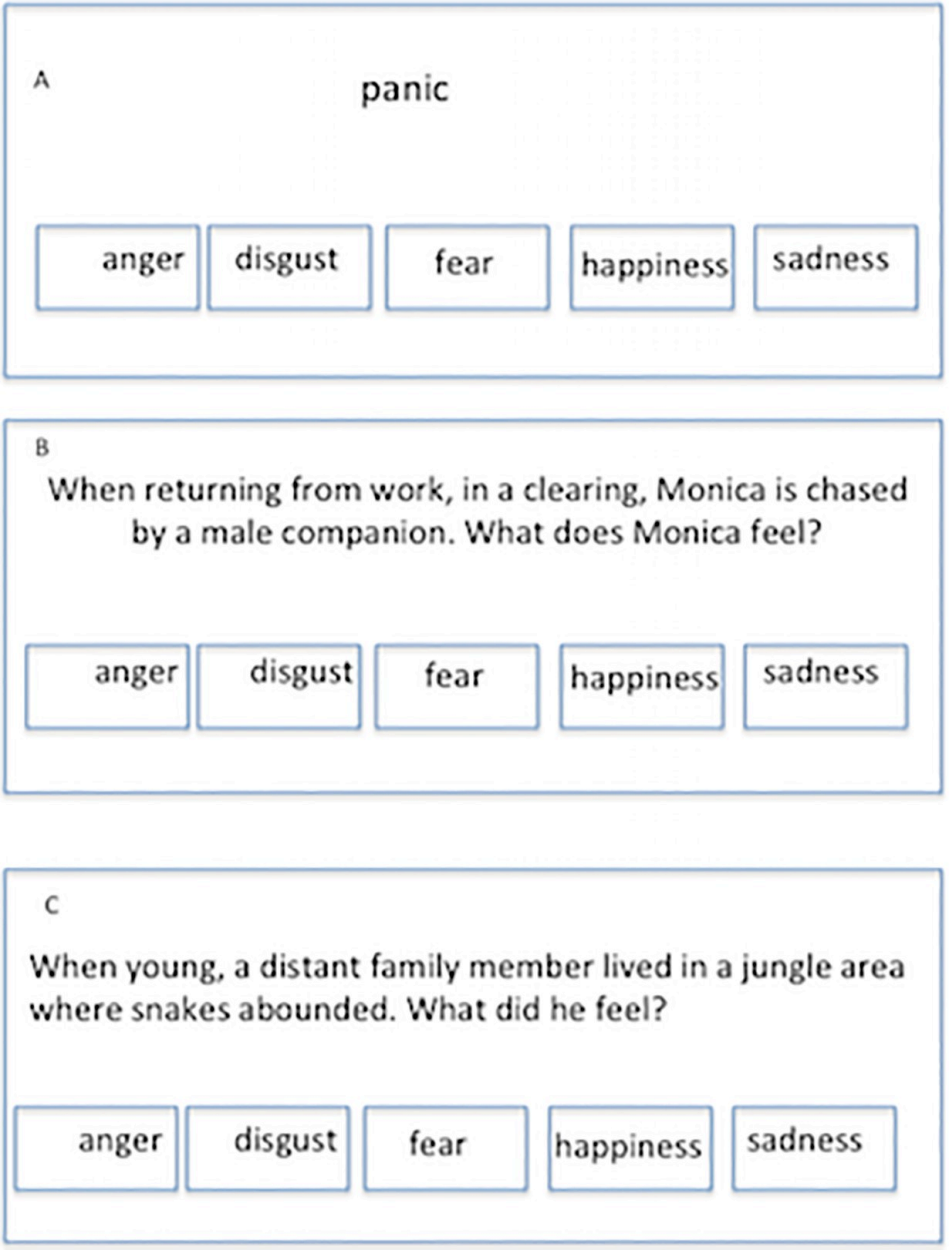
Three FEAR item examples. (A) Emotion Vocabulary item. (B) Close Emotional Situations item. (C) Far Emotional Situations item. Note: Items were written in Spanish, so the translation is an approximation.

The test was implemented on a portable computer. Identification, gender, age, consent, response option and right/wrong answers are stored by the application. Each of the three subtests is composed of forty multiple-choice items, eight for each of the five emotion "families". Each item is composed of a stem and five response options: happiness, sadness, anger, fear, and disgust. Fig 1 shows three item examples.

During item construction, two judges, one for each country, evaluated the content seeking to avoid lexical and situational peculiarities (e.g. words having a slang meaning not contained in the dictionary, scenarios reflecting local particularities). Words and scenarios had to represent emotional prototypes equally understood in both countries.

#### Emotion Vocabulary (EV)

The subtest is composed of items 1–40. Each item stem is an emotion word whose frequency per million is similar in Argentina and in Spain according to CORPES XXI [26]. The participant is asked to choose the response option whose meaning is the closest to that of the target word. An EV item example can be seen in Fig 1A.

#### Close Emotional Situations (CES)

The subtest is composed of items 41–80. Item stems are verbal scenarios that show a character and a close/concrete act, object, moment, and place. Scenarios describe concrete variations of the emotion prototypes. The participant is asked to choose the option that best describes the emotion that would be typical to feel in that situation. A CES item example can be seen in Fig 1B.

#### Far Emotional Situations (FES)

The subtest is composed of items 81–90. Item stems are verbal scenarios that show a far/abstract character, time and situation. Scenarios describe abstract variations of the emotion prototypes. The subject is asked to choose the option that best describes the emotion that would usually be felt in that abstract situation. A FES item example can be seen in Fig 1C.

### Procedure

A university researcher approached participants individually and asked about age, place of residence and first language (inclusion criteria). Individual privacy and anonymity were protected. Following the usual procedures in psychological research, data was aggregated and participants gave informed consent (the computerized test includes a button "I consent" to start the tasks.) The test was applied on a portable computer; administration took between fifteen and thirty minutes. Subjects were debriefed about the study upon completion of the tasks.

### Ethical statement

The participants were treated in accordance with the Helsinki ethical guidelines. The Spanish MINECO responsible committee revised the application (including ethical aspects), and approved the research under Grant PSI2014-52369-P. All participants provided their informed consent twice: verbally, while participants were being invited to take part in the study, and via the computer program. Individual privacy and anonymity were protected.

### Data analysis

Rasch analyses were performed with Winsteps 3.80.1 [24]. Data-model fit was assessed by means of *infit* (an information-weighted form of *outfit*) and *outfit* (calculated by adding the standardized square of residuals after fitting the model over items or subjects to form chi-square-distributed variables). *Infit /outfit* values over 2 are not adequate for the measurement system [24]. Component analyses of residuals are performed by Winsteps 3.80.1 in order to test the unidimensionality assumption. The recommendations are that Rasch measures should account for at least 20% of the total variance [27] and that the unexplained variance in the first contrast be low [28]. As to the assumption of local independence, it was assessed with Yen's Q3 test [29]. High positive correlation of residuals for two items shows that they may be locally dependent. It is usual to compute the correlation matrix of residuals and select the maximum value (Q3,max). However, no single stand-alone critical value exists, and the range of residual correlations values is influenced by various factors, including the number of items [30]. In practical terms, correlations over .70 would be clearly indicative of local dependence (Linacre, 2013).

As to DIF, it was analyzed by testing the standardized difference between item calibrations in two groups across three criteria (gender: 0 = female, 1 = male; age: 0 = below college, 1 = college and over; country: 0 = Spain, 1 = Argentina) with Bonferroni-corrected *alpha* levels; the Rasch-modeled scores from the analysis of all the participants were held constant, providing the conjoint measurement scale in *logit* units. *Welch-t* and *Cohen's d* were calculated to test differences between groups on Rasch scores, before and after removing the seven country-related DIF affected items.

## Results

One (happiness) item got perfect score and therefore its Rasch measure was not estimated. The Rasch analysis of the remaining data indicates good data-model fit for items, *mean infit* was .99 (*SD* = .05) and *mean outfit* was .90 (*SD* = .21). For persons, mean *infit* was 1.00 (*SD* = .16) and *mean outfit* was .90 (*SD* = .50). No item showed *infit/outfit* over 1.5. Eleven persons (less than 3%) showed *outfit* over 2, but none of them showed *infit* over 2. The percentage of variance explained by EK measures was 24.8% and the component analysis of residuals showed that the unexplained variance in the first contrast was 2.7%. *Item reliability* (.97) and *model person reliability* (.80) were adequate. Residual correlations between items were in the range (-.23,.67), with average 0.00. There were no residual correlations over .70. Less than 3 per 1000 residual correlations were in the range .40-.67. Thus, the assumption of local independence for items can be maintained. Table 1 shows the main results of the item analysis.

**Table 1:** EK items: Emotion, score, Rasch Di and SE.

001	HAPPINESS	395	-2.26	.45
002	DISGUST	234	1.99	.11
003	ANGER	380	-.79	.23
004	FEAR	393	-1.91	.39
005	DISGUST	392	-1.77	.36
006	DISGUST	319	.87	.13
007	HAPPINESS	398	-3.19	.71
008	SADNESS	365	-.16	.18
009	ANGER	305	1.09	.12
010	DISGUST	260	1.69	.11
011	SADNESS	385	-1.10	.27
012	HAPPINESS	399	-3.89	1.00
013	HAPPINESS	377	-.64	.22
014	SADNESS	364	-.13	.18
015	DISGUST	328	.72	.13
016	FEAR	294	1.25	.12
017	FEAR	325	.77	.13
018	HAPPINESS	400	—	—
019	ANGER	335	.58	.14
020	HAPPINESS	375	-.55	.21
021	FEAR	117	3.33	.11
022	ANGER	290	1.31	.12
023	DISGUST	118	3.32	.11
024	HAPPINESS	277	1.48	.11
025	FEAR	316	.92	.13
026	SADNESS	267	1.60	.11
027	ANGER	358	.05	.17
028	SADNESS	356	.10	.16
029	HAPPINESS	360	-.01	.17
030	DISGUST	245	1.86	.11
031	FEAR	145	2.99	.11
032	FEAR	393	-1.91	.39
033	SADNESS	339	.50	.14
034	DISGUST	92	3.68	.12
035	ANGER	370	-.34	.19
036	SADNESS	326	.75	.13
037	SADNESS	331	.66	.14
038	FEAR	382	-.91	.25
039	ANGER	98	3.60	.12
040	ANGER	292	1.28	.12
041	ANGER	306	1.08	.12
042	FEAR	379	-.74	.23
043	FEAR	388	-1.34	.30
044	FEAR	353	.18	.16
045	HAPPINESS	395	-2.26	.45
046	ANGER	223	2.11	.11
047	SADNESS	296	1.22	.12
048	DISGUST	213	2.22	.11
049	HAPPINESS	396	-2.49	.51
050	HAPPINESS	397	-2.78	.58
051	FEAR	378	-.69	.22
052	SADNESS	380	-.79	.23
053	ANGER	333	.62	.14
054	ANGER	325	.77	.13
055	ANGER	354	.16	.16
056	DISGUST	272	1.54	.11
057	HAPPINESS	394	-2.07	.42
058	DISGUST	331	.66	.14
059	SADNESS	313	.97	.13
060	DISGUST	369	-.30	.19
061	ANGER	348	.30	.15
062	HAPPINESS	392	-1.77	.36
063	SADNESS	351	.23	.16
064	FEAR	352	.21	.16
065	FEAR	387	-1.26	.29
066	DISGUST	382	-.91	.25
067	FEAR	393	-1.91	.39
068	ANGER	378	-.69	.22
069	DISGUST	370	-.34	.19
070	SADNESS	341	.46	.15
071	ANGER	381	-.85	.24
072	HAPPINESS	397	-2.78	.58
073	SADNESS	372	-.42	.20
074	FEAR	386	-1.18	.28
075	HAPPINESS	391	-1.65	.34
076	DISGUST	360	-.01	.17
077	SADNESS	245	1.86	.11
078	SADNESS	333	.62	.14
079	HAPPINESS	392	-1.77	.36
080	DISGUST	300	1.17	.12
081	DISGUST	347	.33	.15
082	ANGER	323	.80	.13
083	FEAR	371	-.38	.20
084	SADNESS	279	1.45	.11
085	HAPPINESS	394	-2.07	.42
086	ANGER	327	.73	.13
087	ANGER	331	.66	.14
088	DISGUST	350	.26	.16
089	SADNESS	329	.70	.14
090	HAPPINESS	396	-2.49	.51
091	DISGUST	366	-.20	.18
092	FEAR	341	.46	.15
093	HAPPINESS	393	-1.91	.39
094	HAPPINESS	383	-.97	.25
095	SADNESS	330	.68	.14
096	ANGER	360	-.01	.17
097	SADNESS	233	2.00	.11
098	SADNESS	362	-.07	.18
099	DISGUST	286	1.36	.12
100	DISGUST	384	-1.03	.26
101	FEAR	203	2.33	.10
102	FEAR	351	.23	.16
103	SADNESS	373	-.46	.20
104	ANGER	323	.80	.13
105	FEAR	385	-1.10	.27
106	FEAR	351	.23	.16
107	HAPPINESS	393	-1.91	.39
108	DISGUST	317	.90	.13
109	SADNESS	389	-1.44	.31
110	HAPPINESS	390	-1.54	.32
111	ANGER	310	1.02	.12
112	FEAR	386	-1.18	.28
113	HAPPINESS	393	-1.91	.39
114	SADNESS	378	-.69	.22
115	DISGUST	210	2.26	.10
116	HAPPINESS	393	-1.91	.39
117	ANGER	339	.50	.14
118	ANGER	326	.75	.13
119	FEAR	375	-.55	.21
120	DISGUST	382	-.91	.25

**Fig 2:**
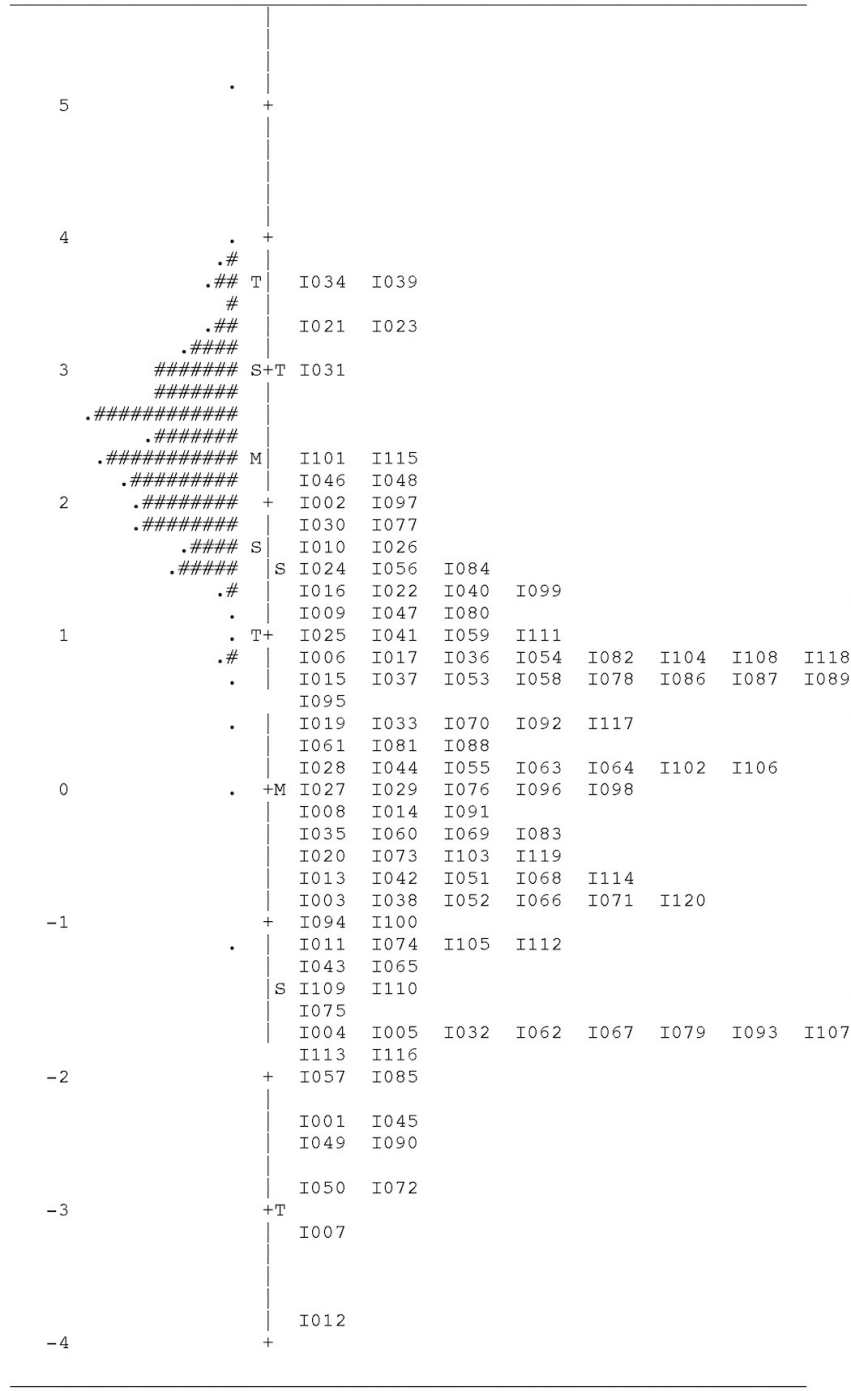
Emotion knowledge: Map of the variable. Note: *M* = mean; *S* = 1 *SD*; *T* = 2 *SD*; each "#" is 4; each "." is 1 to 3.

The map of the variable (or Wright map) can be seen in Fig 2: person measures are on the left while the right side shows item difficulties.

Average person aptitude in *logit* units was 2.36, *SD* = .68, *range* = -1.12 to 5.09. No item showed sex-related DIF, nor were gender differences (impact) found in Rasch measures, *Welch-t* (385) = 1.96, *p* = .051, *d* = -.19 (conventionally coded as 0 = female, 1 = male).

Five items (I20, I24, I29, I56 and I58) showed age-related DIF; two of them, I56 and I58, favored the young group, and thus DIF can be considered as balanced (i.e., a small number of items favored each of the two groups and so it is considered of no consequence). No age-related differences in Rasch measures were found, *Welch-t* (396) = -1.84, *p* = .067, *d* = .18 (coded as 0 = 18–30, 1 = 31–65). Education level was coded as 0 = below college, 1 = college and over. Two items (I24, which favored the less educated group, and I36) showed education-related balanced DIF (i.e., one item favored each of the two groups and so it is considered of no consequence); small-sized education-related differences in Rasch measures were found, *Welch-t* (355) = -2.69, *p* = .008, *d* = .25.

Seven items (I23, I26, I27, I30, I31, I80 and I101) showed country-related DIF, five of which favored the Spanish participants, and two favored the Argentinian ones (I80 and I101). Small significant differences in Rasch measures were found, *Welch-t* (362) = 2.54, *p* = .011, *d* = -.25 (coded as 0 = Spain, 1 = Argentina). Mean scores were 2.45 in Spain and 2.28 in Argentina.

After deleting these seven items, the Rasch analysis of the remaining data showed good fit for items: *mean infit* was .99 (*SD* = .06), and *mean outfit* was .89 (*SD* = .22). For persons, *mean infit* was .99 (*SD* = .15) and *mean outfit* was .89 (*SD* = .48). No item showed *infit/outfit* over 1.5. Twelve persons (3%) showed *outfit* over 2, but none of them showed *infit* over 2.

The percentage of variance explained by EK measures was 22.9% and the component analysis of residuals showed that the unexplained variance in the first contrast was 2.9%. *Item reliability* (.97) and *model person reliability* (.79) were good. Differences in EK scores associated with sex (*Welch-t* (387) = 1.86, *p* = .064, *d* = -.19, conventionally coded as 0 = female, 1 = male), age (*Welch-t* (397) = -1.50, *p* = .14, *d* = .15), educational level (*Welch-t* (353) = -2.19, *p* = .029, *d* = .23) and country (*Welch-t* (374) = .89, *p* = .37, *d* = -.08, coded as 0 = Spain, 1 = Argentina) were non-significant (Bonferroni-corrected).

## Discussion

This study examined whether the EK test showed DIF in two Spanish speaking countries sharing the same language and showing cultural similarities. Based in the conceptual act theory [4], agreement within broad emotional categories for people belonging to a general culture and language was expected, even though some systematic sub-cultural variation in emotional knowledge could also appear.

The generalized validity of the EK test [16] in Argentina was tested with the RM, an implementation of the invariant measurement approach [20, 21]. Results indicated that both fit to the RM and reliability were adequate. There were no significant sex-related or age-related differences in EK. Small differences were found for educational level and country. However, these differences disappeared when the seven country-related DIF affected items were removed. These results are in agreement with the conceptual act theory predictions of a general absence of DIF between the two countries. Only a few items exhibited DIF, probably reflecting some sub-cultural differences. However, this could also be due to *overfitting*: the tendency for statistical models to mistakenly fit sample-specific noise as if it were signal. Minimizing overfitting is needed when the objective is to generalize to new observations that are similar (but not identical) to the ones that have been sampled [31]. This is why we do not recommend deleting these seven items now. If our results are replicated in future studies, then substitution of the seven items must be considered.

Current evidence is sufficient to allow for the EK test to be employed in both Argentina and Spain, in academic or applied settings where individual differences in emotional competence might be relevant. The map of the variable (or Wright map) makes it easy to communicate test results to both academicians and lay people [32]. However, some limitations of our study must be taken into account: the initial validation of the EK scores was carried out on adult samples without disabilities, and so our conclusion is neither applicable to children nor to populations with special needs as, e.g., deaf people. Increasing the number of difficult items is certainly needed in order to reliably assess EK aptitude in high ability samples. We are currently planning to increase the number of high-difficulty emotional vocabulary items.

## Supporting information

S1 File Data file. (TXT)
